# Supplementary material for: Coordination among neighbors improves the efficacy of Zika control despite economic costs
Source: PLoS Negl Trop Dis. 2020 Jun 22;14(6):e0007870. doi: 10.1371/journal.pntd.0007870 (PMC7332071; doi:10.1371/journal.pntd.0007870)
Supplement: S1 Text — These equations are adapted from [37]. (DOCX) [file pntd.0007870.s001.docx]

**Methods Appendix**

We model mosquito populations dynamics, mosquito migration, and disease dynamics using the following discrete-time SIR-type difference equations. Parameters and variables are defined in Tables S1 and S2, respectively. For full model description see [1].

| $M_{j,p,t}=\left( 1-Treat_{p,t} \right)\left( M_{j,p,t-1}\left( 1-\mu\right)+\nu\left( M_{n,p,t-1}+M_{i,p,t-1} \right)\left( 1-\frac{M_{j,p,t-1}}{K} \right)-gM_{j,p,t-1} \right)$ | (1) |
| --- | --- |
| $M_{n,p,t}=\left( M_{n,p,t-1}+\sum_{\forall q\neq p} M_{n,q,t-1}D_{q,p}-\sum_{\forall p\neq q} M_{n,p,t-1}D_{p,q}-rcT_{hm}H_{i,t-1}M_{n,p,t-1} \right)\left( 1-\mu\right)+gM_{j,p,t-1}$ | (2) |
| $M_{i,p,t}=\left( M_{i,p,t-1}+\sum_{\forall q\neq p} M_{i,q,t-1}D_{q,p}-\sum_{\forall p\neq q} M_{i,p,t-1}D_{p,q}+rcT_{hm}H_{i,t-1}M_{n,p,t-1} \right)\left( 1-\mu\right)$ | (3) |
| $H_{s,t}=H_{s,t-1}-H_{s,t-1}rcT_{mh}\sum_{\forall p} M_{i,p,t-1}$ | (4) |
| $H_{i,t}=H_{i,t-1}+H_{s,t-1}rcT_{mh}\sum_{\forall p} M_{i,p,t-1}-\gamma H_{i,t-1}$ | (5) |
| $H_{r,t}=H_{r,t-1}+\gamma H_{i,t-1}$ | (6) |

**References**

1. Schwab SR, Stone CM, Fonseca DM, Fefferman NH. The importance of being urgent: The impact of surveillance target and scale on mosquito-borne disease control. Epidemics. 2017; doi:10.1016/j.epidem.2017.12.004
