# Supplementary material for: Coordination among neighbors improves the efficacy of Zika control despite economic costs
Source: PLoS Negl Trop Dis. 2020 Jun 22;14(6):e0007870. doi: 10.1371/journal.pntd.0007870 (PMC7332071; doi:10.1371/journal.pntd.0007870)
Supplement: S1 Table — (DOCX) [file pntd.0007870.s002.docx]

**Table S1. Parameter definitions and values used in model simulations**

| **Parameter** | **Value(s)** | **Definition** |
| --- | --- | --- |
| *Treat* | 0 = untreated patch  1 = treated with no coordination costs  0.9 = treated with reduced efficacy due to coordination costs (nearest neighbor)  0.8 = treated with reduced efficacy due to coordination costs (second nearest neighbor) | Fraction of larva killed by control in a patch |
| *µ* | 1/20 | Per capita death rate of mosquitoes  (after density-independent mortality) |
| *ν* | 3 | Per capita birth rate of mosquitoes (after density-independent mortality) |
| *K* | 350 | Carrying capacity of juvenile mosquitoes in each patch |
| *g* | 1/10 | Growth rate of mosquitoes from juvenile to adult |
| *D* | $\sum_{\forall q\neq p} D_{p,q}= 0.1$ | Matrix of mosquito dispersal probabilities between patches |
| *r* | .3 | Biting rate |
| *c* | 0.003 | Scaling constant (to enable reasonable pace of outbreak amid a ubiquitous human population) |
| *T* | *T_mh_* = 0.08  *T_hm_* = 0.07 | Transmission probabilities per bite from  mosquitoes to humans (T_mh_) and humans to mosquitoes (T_hm_) |
| *γ* | 1/4 | Recovery rate of humans |
| *Thresh* | 1 = Epidemiological surveillance, high sensitivity  5 = Epidemiological surveillance, low sensitivity  0.1 = Demographic surveillance, high sensitivity  0.5 = Demographic surveillance, low sensitivity | Threshold for triggering larval control in a patch |
