# Supplementary material for: Coordination among neighbors improves the efficacy of Zika control despite economic costs
Source: PLoS Negl Trop Dis. 2020 Jun 22;14(6):e0007870. doi: 10.1371/journal.pntd.0007870 (PMC7332071; doi:10.1371/journal.pntd.0007870)
Supplement: S2 Table — (DOCX) [file pntd.0007870.s003.docx]

**Table S2. Variables used in model equations.**

| **Variable** | **Definition** |
| --- | --- |
| M_j_ | Number of juvenile (pre-adult) mosquitoes |
| M_n_ | Number of adult naïve (uninfected) female mosquitoes |
| M_i_ | Number of adult infected female mosquitoes |
| H_s_ | Number of susceptible humans |
| H_i_ | Number of infected humans |
| H_r_ | Number of recovered humans |
| *p* and *q* | Patch identifiers |
| *t* | Day identifier |
