# Supplementary figures and images for: Coordination among neighbors improves the efficacy of Zika control despite economic costs
Source: PLoS Negl Trop Dis. 2020 Jun 22;14(6):e0007870. doi: 10.1371/journal.pntd.0007870 (PMC7332071; doi:10.1371/journal.pntd.0007870)

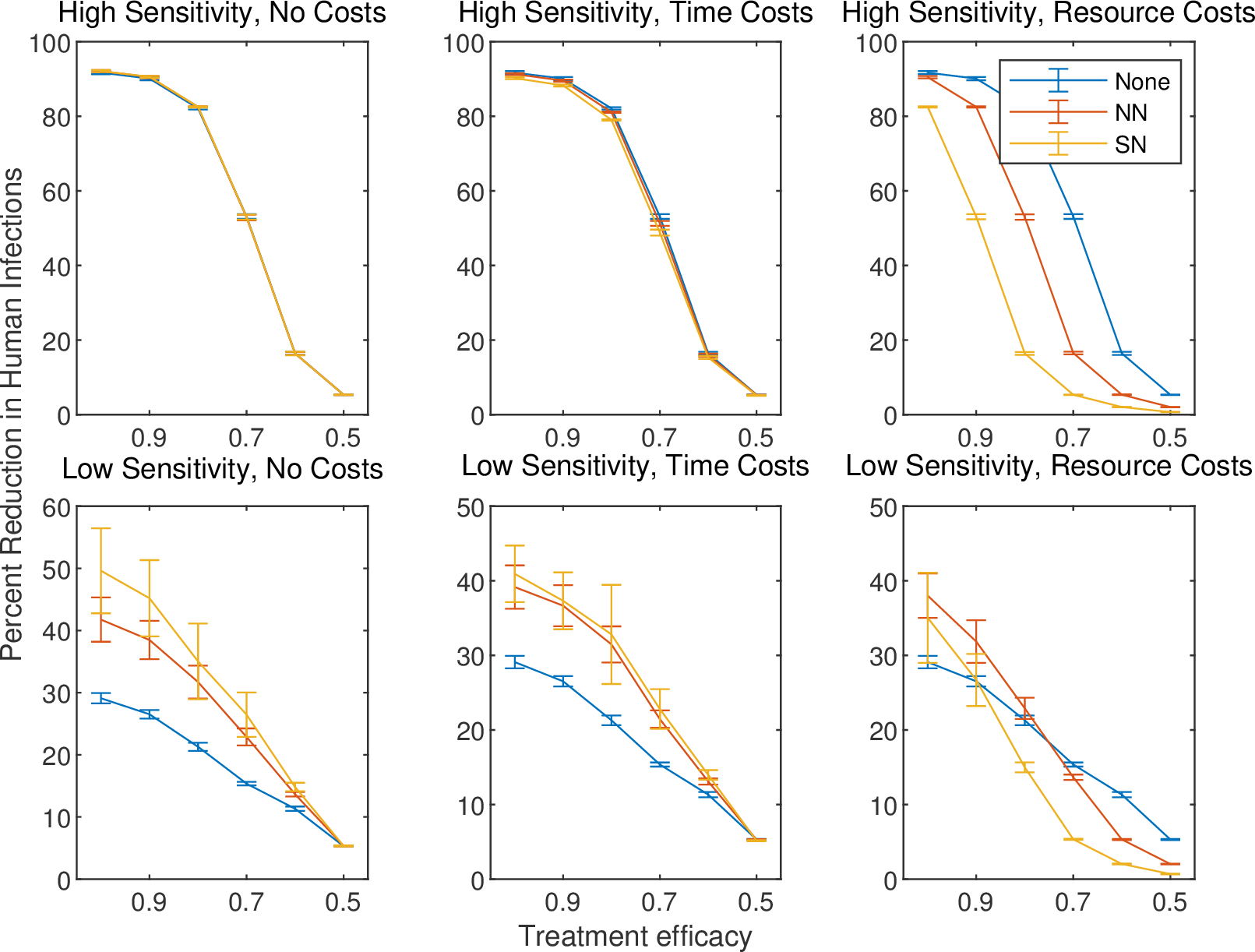

Supplement: S1 Fig — Blue lines (None) show the reduction in human infections when larval control is enacted only in patches that exceed the surveillance threshold. Red lines (NN) show the result when control occurs in the triggered patch and its nearest neighbors. Yellow lines (SN) show the result when control occurs in a triggered patch's nearest neighbors and second nearest neighbors. At high surveillance sensitivity, the efficacy of larvicide treatment has no impact on the benefits of coordination among neighbors. At low sensitivity, the efficacy of the larvicide treatment does affect the benefits of coordination but only when coordination reduces the treatment efficacy in each patch (right panels). (TIF) [file pntd.0007870.s004.tif]

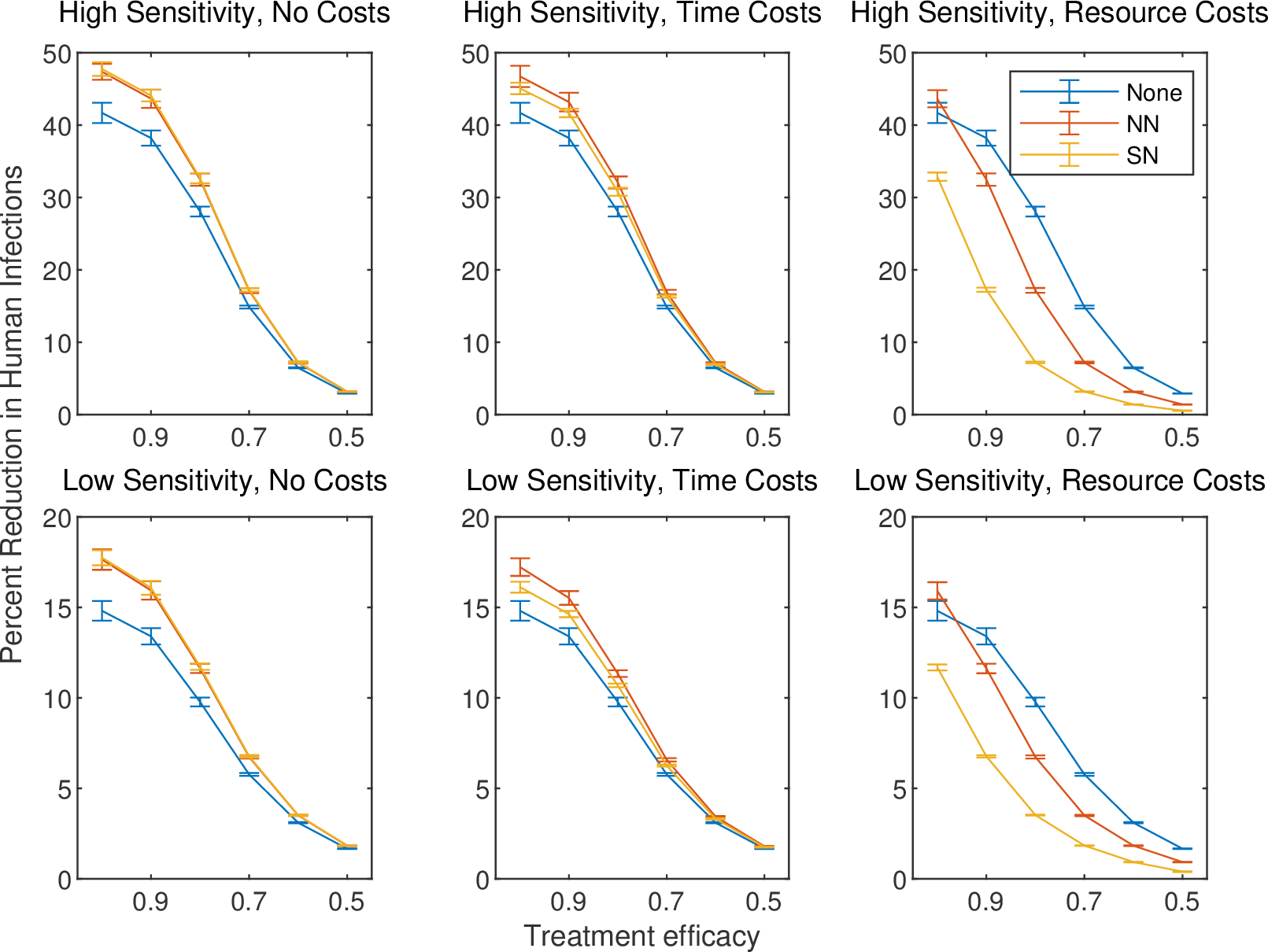

Supplement: S2 Fig — Blue lines (None) show the reduction in human infections when larval control is enacted only in patches that exceed the surveillance threshold. Red lines (NN) show the result when control occurs in the triggered patch and its nearest neighbors. Yellow lines (SN) show the result when control occurs in a triggered patch's nearest neighbors and second nearest neighbors. Without costs, coordination with nearest neighbors improves infection outcomes. However, unless larvicide has very high initial efficacy, any reduction in efficacy resulting from coordination results in worse outcomes than not coordinating. (TIF) [file pntd.0007870.s005.tif]
