# Supplementary material for: Coordination among neighbors improves the efficacy of Zika control despite economic costs
Source: PLoS Negl Trop Dis. 2020 Jun 22;14(6):e0007870. doi: 10.1371/journal.pntd.0007870 (PMC7332071; doi:10.1371/journal.pntd.0007870)
Supplement: Attachment — Submitted filename: plos ntd response to reviewer comments.docx [file pntd.0007870.s006.docx]

Responses to Reviewers’ Comments

**Reviewer #2: -Are the objectives of the study clearly articulated with a clear testable hypothesis stated?
Yes.
-Is the study design appropriate to address the stated objectives?
Yes
-Is the population clearly described and appropriate for the hypothesis being tested?
Yes.
-Is the sample size sufficient to ensure adequate power to address the hypothesis being tested?
Yes.
-Were correct statistical analysis used to support conclusions?
n/a
-Are there concerns about ethical or regulatory requirements being met?
No.
-Can you describe where the parameter values were derived from or the reasoning for the chosen values?
- The parameter assumptions about proportions of larvae killed in treated/untreated areas may not be accurate. If we are saying that it is not possible to treat all larval habitats because they are cryptic and/or ephemeral, then treatment doesn’t make a difference to the larval survival or at least will only ever be partial.**

We agree that larvicide treatment may not be 100% effective within each patch. However, even imperfect treatment will reduce larval survival in treated patches. To examine how our assumptions about treatment efficacy affect our results, we conducted a sensitivity analysis of the treatment efficacy parameter. We added the results of this sensitivity analysis to the Results section of the manuscript and to Figures S1 and S2 of the Supplementary Materials.

**-lines 139-152: are there any examples of locations/control programs that use the epidemiologic-based surveillance that you describe, in real time? This sounds very resource-intensive and I would be surprised if anyone is able to do it.**

We agree that epidemiological-based surveillance is a resource-intensive task. Our goal in modeling this type of surveillance is to assess whether such an approach would be useful. **Reviewer #2: Results
-Does the analysis presented match the analysis plan?
Yes.
-Are the results clearly and completely presented?
Yes.
-Are the figures (Tables, Images) of sufficient quality for clarity?
Yes.

Reviewer #2: Conclusions
-Are the conclusions supported by the data presented?
-Are the limitations of analysis clearly described?
No. Please elaborate on the limitations of choosing single parameter values (versus a range) and limitations related to the assumptions made in the study.**

We have added a table to the Methods section listing all of our model assumptions. We have also added Figures S1 and S2 to the Supplementary Materials, where we examine model outcomes under a range of values for the larvicide efficacy parameter. We also added several sentences to the Discussion section (lines 345-250), elaborating on how our assumptions about treatment efficacy affect our results.

**-Do the authors discuss how these data can be helpful to advance our understanding of the topic under study?
Yes.
-Is public health relevance addressed?
Yes.

Reviewer #2: -line 70: Guillain-Barré has an accent on the ‘e’**

Thank you for pointing out this typo. We have corrected it in the manuscript.

**-line 75-76: this sentence is confusing; there is no Zika vaccine yet and there are some dengue vaccines with limited availability**

We have revised this sentence to clarify that there is no vaccine yet for Zika and that the dengue vaccine has limited availability/efficacy.

**-line 78: Albopictus is not capitalized, sentence thought is incomplete (immature what?)**

We have corrected this typo and revised this sentence to clarify that it refers to immature mosquito larvae. **Reviewer #1: The authors present an interesting study modeling larviciding activities in different contexts on surveillance. I found the article to be well written and well described.

1.      The authors need a limitation section in the discussion, with points about the assumptions their model makes. Equal spatial distribution, finding all the breeding sites, etc.**

We added a table to the Methods section listing all the model assumptions. We also added several sentences to the Discussion (lines 345-350) to describe how our assumptions about larvicide efficacy affect our results.

**2.      The article would benefit greatly from a visual representation of the model that is being tested.**

Thank you for this suggestion. We have added a new figure (called Figure 1) to the Methods section to provide a visual representation of the model. We believe this new figure makes the structure of the model and its assumptions much clearer to the reader.

**3.      The abstract references surveillance, the title references control. Can the authors standardize those? I see that the paper is examining coordinated control efforts in different contexts of surveillance. But currently that is a bit confusing.**

We revised the abstract to clarify that surveillance information is used to decide when to enact larval control and that coordination among neighboring patches is in the timing of larval control (when one patch’s surveillance threshold is exceeded, neighboring patches are treated as well).

**4.      In the abstract line 36 can the authors spell out SIR?**

We have revised line 36 of the Abstract and line 118 of the Introduction to define SIR where the term is first introduced.

**5.      Line 90. Perhaps revise to remove hanging preposition; “migration of biting adults from untreated patches may compensate for mortality in treated patches…”**

We have revised this sentence as suggested.

**6.      Line 118. Spell out first instance of SIR.**

We have revised this line to define SIR where it is first introduced.

**7. Need instructions in the manuscript for accessing the data.**

We have added a Data Availability Statement at the end of the manuscript stating that the model code will be made available in the Dryad data repository upon acceptance. **Reviewer #2: This paper considers the spread of Aedes aegypti and Aedes albopictus between controlled and non-controlled areas and asks how coordination between these areas might benefit disease control even under conditions where this coordination is costly in terms of time or resources.

Lines 84-94 describe an interesting scenario and give good reason for asking the proposed research question, but it isn’t clear to me that this scenario is applicable to Aedes aegypti or Aedes albopictus. The adults of these species have a very short flight span, and it seems that only under rare occasions would these adults be traveling between controlled and non-controlled patches.**

We agree that the short flight range of *Aedes* mosquitoes makes them unlikely to travel between cities. We have revised the Introduction to better clarify the spatial scale of the model. We revised lines 89-92 and 104 to state that, as with previous work (Schwab et al 2017), the patches in our model represent neighborhoods or areas within a city rather than entire cities. We also revised line 92 of the Introduction and lines 132-134 of the Methods to clarify that mosquitoes can only recolonize adjacent patches, not any patch in the landscape.

**If we imagine control within a city, that control is already being coordinated within the city, and these Aedes species are unlikely to travel outside of the city in their adult stage. It is hard to imagine patches of adult migration range that would be under different treatment schedules.**

We have revised the Introduction to more clearly describe existing mosquito control strategies (lines 82-94). While some mosquito control efforts are city-wide, more localized approaches, such as chemical treatment of household water storage containers or removal of potential larval habitat by residents, are commonly used as well (Alvarado-Castro et al 2017, Eisen et al 2009, Fonseca et al 2013).

**Even if we think about the edges of different neighborhoods within a city that may be treated a different times for larval control, we would have to imagine a scenario under which there is no adulticide being applied or that it is being applied infrequently enough that there is a window of time during which the adults would travel between these neighborhoods and could find untreated pools in which to lay eggs. It’s not clear that those windows of time are that great (a few days at most?) within an average city in these endemic areas.**

Larvicide and source reduction are often used for mosquito control without adulticide (Fonseca et al. 2013; George et al. 2015). Even when adulticide is used, it is unlikely to be 100% effective (Eisen et al. 2009). Although we acknowledge that larvicide and adulticide are frequently used together as part of integrated vector management, assuming no adulticide in the present model allows us to isolate the effects of different larval control regimens on disease outcomes. **This paper’s hypothesis would be better supported with some description of control plans in endemic areas and more background on control in general so that the reader may better evaluate the likelihood of the situations described.**

We have added a more detailed description of the types of mosquito control plans that are currently being used in arbovirus-endemic areas (lines 82-94 of the Introduction). **Many places use larval surveillance methods (generally cheaper and less labor-intensive than adult surveillance)…. would the results change if larval surveillance and larval control were the focus? Can you further elaborate on this limitation?**

We have added a sentence to the Discussion (lines 358-361) to mention previous work (Schwab et al 2017) that examined the effects of basing control decisions on surveillance of larval or adult mosquito abundance as well as surveillance of human or mosquito infections. This previous work found that the type of surveillance used to inform vector control decisions does influence infection outcomes. In the present work, for simplicity, we chose to examine only two types of surveillance (total adult mosquitoes and infected adult mosquitoes), since our main focus was on understanding the effect of coordination costs. We chose adult abundance rather than larval abundance because previous work (Schwab et al 2017) found that at high sensitivity it was the most effective surveillance target for reducing infections.
